# 30 years in service, 1996–2026: Infectious Diseases Working Party (AGIHO) of the German Society of Hematology and Medical Oncology (DGHO)

**DOI:** 10.1007/s00277-026-07194-8

**Published:** 2026-08-03

**Authors:** Oliver A. Cornely, Georg Maschmeyer, Wolfgang Hiddemann, Nico Bekaan, Nicola Giesen, Cornelia Lass-Flörl, Sibylle C. Mellinghoff, Verena Petzer, Jon Salmanton-García, Michael Sandherr, Enrico Schalk, Xaver Schiel, Martin Schmidt-Hieber, Danila Seidel, Jannik Stemler, Daniel Teschner, Laura N. Walti, Christina T. Rieger

**Affiliations:** 1 Faculty of Medicine and University Hospital Cologne, Institute of Translational Research, Cologne Excellence Cluster on Cellular Stress Responses in Aging-Associated Diseases (CECAD), University of Cologne, Herderstr. 52 , 50931 Cologne, Germany; 2 Faculty of Medicine, Department I of Internal Medicine, Center for Integrated Oncology Aachen Bonn Cologne Duesseldorf (CIO ABCD) and Excellence Center for Medical Mycology (ECMM), University Hospital Cologne, University of Cologne, Cologne, Germany; 3 Partner site Bonn-Cologne, German Centre for Infection Research (DZIF), Cologne, Germany; 4 Clinical Trials Centre Cologne (ZKS Köln), University of Cologne, Cologne, Germany; 5 German Society of Hematology and Medical Oncology (DGHO) and Charité University Medicine Berlin, Campus Benjamin Franklin; Department of Hematology, Oncology and Tumor, Charité University Medicine Berlin, Berlin, Germany; 6 Department of Medicine III, LMU Klinikum, Munich, Germany; 7 Department of Hematology, Oncology and Palliative Care; Robert Bosch Centre for Tumour Diseases (RBCT), Robert Bosch Hospital, Stuttgart, Germany; 8 Institute of Hygiene and Medical Microbiology, ECMM Excellence Diamond Centre of Medical Mycology, Medical University of Innsbruck, Innsbruck, Austria; 9 University Hospital of Internal Medicine V Hematology and Oncology, Medical University of Innsbruck, Innsbruck, Austria; 10 German Society of Hematology and Medical Oncology (DGHO), Berlin, Germany; 11 Medical Faculty, Department of Hematology, Oncology, Cell and Radiation Therapy, Otto von Guericke University Magdeburg, Magdeburg, Germany; 12 MVZ Penzberg – Filialpraxis Weilheim, Weilheim, Germany; 13 Clinic for Hematology, Oncology, Pneumology, Nephrology and Diabetology, Medical University Lausitz – Carl Thiem (MUL-CT), Cottbus, Germany; 14 Department of Internal Medicine II, University Hospital Würzburg, Würzburg, Germany; 15 Department of Infectious Diseases, Inselspital, Bern University Hospital, University of Bern, Bern, Switzerland; 16 Hematology Oncology Germering, Landsberger Str. 27, 82110 Germering, Germany

**Keywords:** Guidelines, Leukemia, Lymphoma, Solid tumor, Infection, Vaccine

## Abstract

The Infectious Diseases Working Party (AGIHO) was established in 1996 as one of the subgroups of the German Society of Hematology and Medical Oncology (DGHO). Marking its 30th anniversary this year, the AGIHO reflects on a period of significant achievement and growth. Beyond its core mission of developing evidence-based clinical practice guidelines for the prevention, diagnosis, and management of infections in patients with cancer, the AGIHO has evolved into a vital platform for clinical trials, collaborative research, and postgraduate medical education. To ensure long-term sustainability and foster emerging talent, “Young AGIHO” was launched in 2023. This initiative aims to strengthen networking among early career physicians specializing in hematology and oncology with a strong focus on infectious diseases. The working party has also expanded its geographical footprint by integrating colleagues from Austria and Switzerland, thereby enhancing its international presence. Through the publication of guidelines in high-ranking international journals, AGIHO visibility has increased significantly. Representatives of the working party now serve as experts for scientific and health policy committees both nationally and internationally. As one of the DGHO's largest and most active subgroups, the AGIHO is ideally positioned to address future challenges in the field.

## Introduction

The prevention, early diagnosis, effective empirical or pre-emptive antimicrobial treatment of infections have become essential cornerstones in the management of patients with hematological malignancies. In acute myeloid leukemia (AML), particularly in advanced age, improvement of morbidity and of prognosis has largely been achieved by better antifungal strategies, besides more effective antiemetic prophylaxis and platelet transfusions [1]. Along with highly effective antimicrobial agents used for prophylaxis and interventional treatment, increasing rates of multi-resistance, e.g., to broad-spectrum antibacterial drugs such as carbapenems and to triazole antifungals, has called to action among hematologists, oncologists, hematopoietic cell transplant (HCT) experts, infectious disease (ID) and infection control experts, so that antimicrobial stewardship and effective multi-disciplinary co-operation have become of paramount importance [2, 3].

However, the increasing diversity of cancer treatment approaches, using immunotherapies aiming at T-cell activation as well as multi-tyrosine kinase inhibitors, monoclonal antibodies including bispecific T-cell engagers and combination therapies, as well as the introduction of new concepts for allogeneic HCT and graft-*versus*-host management, have resulted in a much broader diversity of immunosuppression than historically known. The integration of ID into clinical hematology and HCT have emerged as an essential demand [4]. The knowledge of infectious complications associated with different types of immunomodulatory and immunosuppressive therapy has led to a broad range of specific, evidence-based clinical guidelines from the Infectious Diseases Working Party (“Arbeitsgemeinschaft Infektionen in der Hämatologie und Onkologie [AGIHO]”) of the German Society for Hematology and Medical Oncology (DGHO) and other societies, which are updated on a regular basis and have become part of daily clinical practice [5, 6].

## Founding history

After successful multicenter studies on antimicrobial therapy in patients with acute leukemia by the Paul Ehrlich Society for Chemotherapy (“Paul Ehrlich Gesellschaft für Chemotherapie [PEG]”), two of the principal investigators, Wolfgang Hiddemann and Georg Maschmeyer, decided using this momentum to establish a standardized strategy for antimicrobial management in patients with AML undergoing intensive induction therapy. With the help of Xaver Schiel, a series of workshops was initiated using the German AML Cooperative Group (AMLCG) as cooperative partner. Several guidelines were developed and clinically applied. In 1996, the DGHO accepted an antimicrobial working group as an official organ of the DGHO and the AGIHO was established, and Wolfgang Hiddemann and Georg Maschmeyer were elected as founding chairmen. Since then, the AGIHO has published evidence-based guidelines for prophylaxis, diagnosis and treatment of infections in patients with hematological or oncological diseases and HCT recipients.

Since its inception, the ID Working Party reached out to Austria and Switzerland strengthening the D-A-CH (Germany, Austria, Switzerland) perspective and increasing applicability of AGIHO recommendations across different healthcare systems. By connecting Austrian and Swiss centers within AGIHO initiatives, consensus building and harmonization, cross-border knowledge transfer was measurably enhanced. Apart from hematology, oncology, and HCT experts, AGIHO members represent adult and pediatric clinical ID, microbiology, virology, intensive care, and radiology allowing pragmatic, clinically oriented guideline development. Examples for fruitful collaboration are the strong Austrian contribution to quality assured diagnostics and stewardship, and the Swiss model of general ID specialists providing expert care for severely immunocompromised patients.

AGIHO members regularly consult with the Ministries of Health with topics ranging from incipient epidemiologic developments to vaccine hesitancy, to antimicrobial resistance, to commenting on developing regulations and laws.

## Current fields of activity

### Young AGIHO

The Young AGIHO (yAGIHO) was founded at the joint annual conference of DGHO, OeGHO (Austrian Society for Hematology and Medical Oncology) and SGH + SSH/SGMO (Swiss Society of Hematology/Swiss Society of Medical Oncology) in 2023. It represents young physicians and medical students at the beginning of their careers. The major aim is including physicians-in-training in the active work of the AGIHO and to interconnect between centers and disciplines. yAGIHO members are represented in all guideline panels and contribute to registry studies and clinical trials.

Furthermore, yAGIHO fosters active discussions on specialization training and clinical decision making from everyday life of residents and fellows, particularly in regular virtual events with case presentations and journal clubs.

### Education and training

AGIHO has been hosting a 2-day training course annually since 2003. This educational event is a major training and networking platform for senior clinicians and young scientists alike. Members of the AGIHO steering committee and international guest speakers share their expertise and the latest knowledge on diagnostics, prophylaxis, and treatment of infections in hematology and oncology. AGIHO presents at all major national conferences with bespoke symposia, and the joint annual conference hosts a variety of very well attended AGIHO sessions.

### Guidelines

**Fig. 1:**
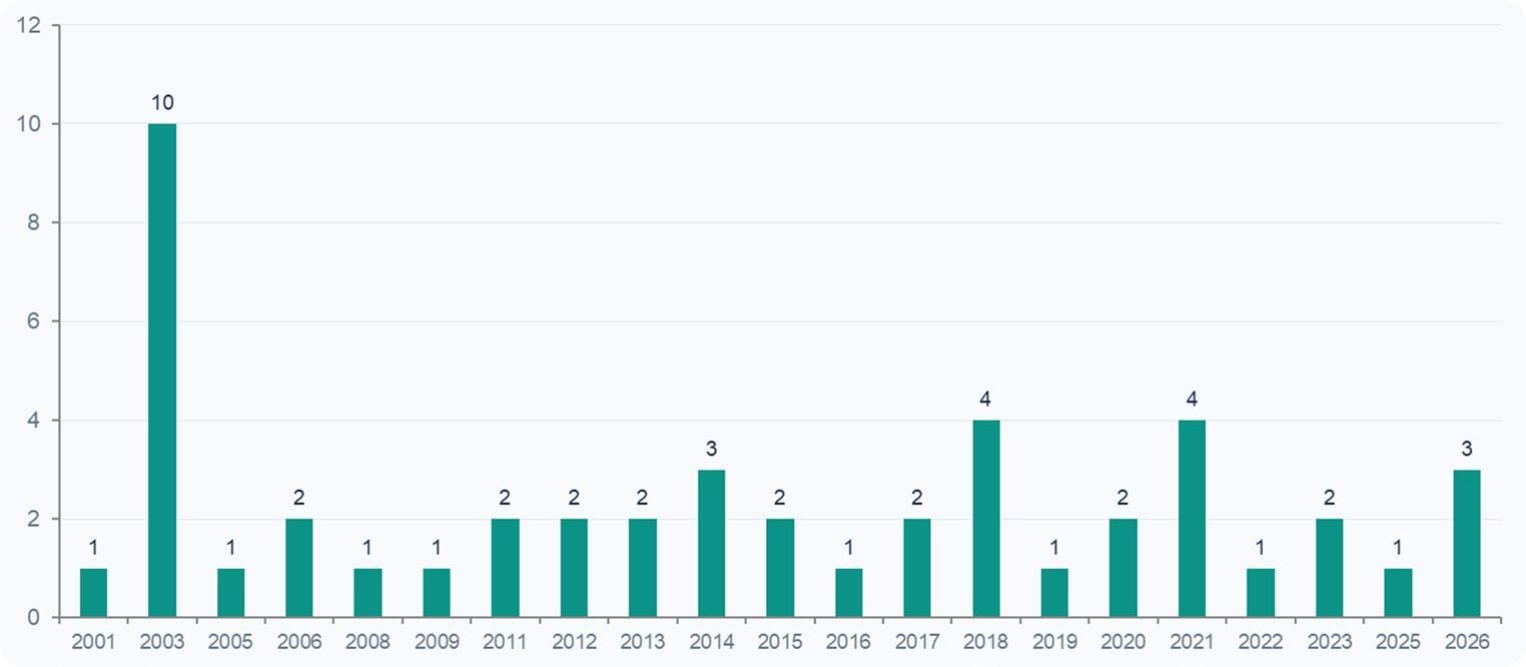
Publication record of the ID working party

AGIHO has published 48 guidelines and research papers on all fields of infection in cancer patients that collectively received 1,273 citations (Table 1), corresponding to an average of 26 citations per paper. AGIHO guidelines are open access and regularly updated, as reflected by the continuous flow of publications, averaging about one journal publication every six months (Figure 1). Condensed excerpts of the guidelines are published on www.dgho.de in German and English language. Since 2025 the platform www.medicalguideline.org is used to support collaborative work with a standardized format, integrated literature management, and artificial intelligence assisted tools.

**Table 1:** Guidelines of the ID working party

Year	Title
2026	Anti-infective Vaccination Strategies in Patients with Hematological Malignancies or Solid Tumors – 2025 Update of the Guideline of the Infectious Diseases Working Party (AGIHO) of the German Society for Hematology and Medical Oncology (DGHO) [7]
2026	Evidence-based AGIHO guideline update on prophylaxis of infectious complications with granulocyte-stimulating factors (G-CSF) for the treatment of adult patients with cancer [8]
2026	Prevention, diagnosis and management of community acquired respiratory virus infections including COVID-19 in patients with cancer: 2025 updated evidence-based guideline of the infectious diseases working party (AGIHO) of the German society for hematology and medical oncology (DGHO) [9]
2026	CNS infections in patients with hematological or oncological diseases (including cellular therapies) − 2024 update of the guideline of the Infectious Diseases Working Party (AGIHO) of the German Society of Hematology and Medical Oncology (DGHO) [10]
2025	2024 update of the AGIHO guideline on diagnosis and empirical treatment of fever of unknown origin (FUO) in adult neutropenic patients with solid tumours and hematological malignancies [11]
2023	Primary prophylaxis of invasive fungal diseases in patients with haematological malignancies: 2022 update of the recommendations of the Infectious Diseases Working Party (AGIHO) of the German Society for Haematology and Medical Oncology (DGHO) [12]
2023	AGIHO guideline on evidence-based management of COVID-19 in cancer patients: 2022 update on vaccination, pharmacological prophylaxis and therapy in light of the omicron variants [13]
2022	Management of herpesvirus reactivations in patients with solid tumours and hematologic malignancies: update of the Guidelines of the Infectious Diseases Working Party (AGIHO) of the German Society for Hematology and Medical Oncology (DGHO) on herpes simplex virus type 1, herpes simplex virus type 2, and varicella zoster virus [14]
2021	Primary prophylaxis of bacterial infections and Pneumocystis jirovecii pneumonia in patients with hematologic malignancies and solid tumors: 2020 updated guidelines of the Infectious Diseases Working Party of the German Society of Hematology and Medical Oncology (AGIHO/DGHO) [15]
2021	2021 update of the AGIHO guideline on evidence-based management of COVID-19 in patients with cancer regarding diagnostics, viral shedding, vaccination and therapy [16]
2021	Prophylaxis, diagnosis and therapy of infections in patients undergoing high-dose chemotherapy and autologous haematopoietic stem cell transplantation. 2020 update of the recommendations of the Infectious Diseases Working Party (AGIHO) of the German Society of Hematology and Medical Oncology (DGHO) [17]
2021	Central venous catheter-related infections in hematology and oncology: 2020 updated guidelines on diagnosis, management, and prevention by the Infectious Diseases Working Party (AGIHO) of the German Society of Hematology and Medical Oncology (DGHO) [18]
2020	Evidence-based management of COVID-19 in cancer patients: Guideline by the Infectious Diseases Working Party (AGIHO) of the German Society for Haematology and Medical Oncology (DGHO) [19]
2020	Treatment of invasive fungal diseases in cancer patients-Revised 2019 Recommendations of the Infectious Diseases Working Party (AGIHO) of the German Society of Hematology and Oncology (DGHO) [20]
2019	Management of sepsis in neutropenic cancer patients: 2018 guidelines from the Infectious Diseases Working Party (AGIHO) and Intensive Care Working Party (iCHOP) of the German Society of Hematology and Medical Oncology (DGHO) [21]
2018	Diagnosis of invasive fungal diseases in haematology and oncology: 2018 update of the recommendations of the infectious diseases working party of the German society for hematology and medical oncology (AGIHO) [22]
2018	Anti-infective vaccination strategies in patients with hematologic malignancies or solid tumors-Guideline of the Infectious Diseases Working Party (AGIHO) of the German Society for Hematology and Medical Oncology (DGHO) [23]
2018	Primary prophylaxis of invasive fungal infections in patients with haematological malignancies: 2017 update of the recommendations of the Infectious Diseases Working Party (AGIHO) of the German Society for Haematology and Medical Oncology (DGHO) [24]
2018	Diagnosis and management of gastrointestinal complications in adult cancer patients: 2017 updated evidence-based guidelines of the Infectious Diseases Working Party (AGIHO) of the German Society of Hematology and Medical Oncology (DGHO) [25]
2017	Diagnosis and empirical treatment of fever of unknown origin (FUO) in adult neutropenic patients: guidelines of the Infectious Diseases Working Party (AGIHO) of the German Society of Hematology and Medical Oncology (DGHO) [26]
2016	CNS infections in patients with hematological disorders (including allogeneic stem-cell transplantation)-Guidelines of the Infectious Diseases Working Party (AGIHO) of the German Society of Hematology and Medical Oncology (DGHO) [27]
2015	Antiviral prophylaxis in patients with solid tumours and haematological malignancies–update of the Guidelines of the Infectious Diseases Working Party (AGIHO) of the German Society for Hematology and Medical Oncology (DGHO) [28]
2015	Diagnosis and antimicrobial therapy of lung infiltrates in febrile neutropenic patients (allogeneic SCT excluded): updated guidelines of the Infectious Diseases Working Party (AGIHO) of the German Society of Hematology and Medical Oncology (DGHO). [29]
2014	Management of sepsis in neutropenic patients: 2014 updated guidelines from the Infectious Diseases Working Party of the German Society of Hematology and Medical Oncology (AGIHO) [30]
2014	Prophylaxis of infectious complications with colony-stimulating factors in adult cancer patients undergoing chemotherapy-evidence-based guidelines from the Infectious Diseases Working Party AGIHO of the German Society for Haematology and Medical Oncology (DGHO) [31]
2014	Treatment of invasive fungal infections in cancer patients-updated recommendations of the Infectious Diseases Working Party (AGIHO) of the German Society of Hematology and Oncology (DGHO) [32]
2013	Primary prophylaxis of bacterial infections and Pneumocystis jirovecii pneumonia in patients with hematological malignancies and solid tumors: guidelines of the Infectious Diseases Working Party (AGIHO) of the German Society of Hematology and Oncology (DGHO) [33]
2013	Diagnosis and management of gastrointestinal complications in adult cancer patients: evidence-based guidelines of the Infectious Diseases Working Party (AGIHO) of the German Society of Hematology and Oncology (DGHO) [34]
2012	Antimicrobial therapy of febrile complications after high-dose chemotherapy and autologous hematopoietic stem cell transplantation–guidelines of the Infectious Diseases Working Party (AGIHO) of the German Society of Hematology and Oncology (DGHO) [35, 36]
2012	Diagnosis of invasive fungal infections in hematology and oncology–guidelines from the Infectious Diseases Working Party in Haematology and Oncology of the German Society for Haematology and Oncology (AGIHO) [36]
2009	Treatment of invasive fungal infections in cancer patients – Recommendations of the Infectious Diseases Working Party (AGIHO) of the German Society of Hematology and Oncology (DGHO) [37]
2008	Central venous catheter-related infections in hematology and oncology: guidelines of the Infectious Diseases Working Party (AGIHO) of the German Society of Hematology and Oncology (DGHO) [38]
2006	Management of sepsis in neutropenia: guidelines of the infectious diseases working party (AGIHO) of the German Society of Hematology and Oncology (DGHO) [39]
2006	Antiviral prophylaxis in patients with haematological malignancies and solid tumours: Guidelines of the Infectious Diseases Working Party (AGIHO) of the German Society for Hematology and Oncology (DGHO) [40]
2005	Antimicrobial prophylaxis in allogeneic bone marrow transplantation. Guidelines of the infectious diseases working party (AGIHO) of the German society of haematology and oncology [41]
2003	Antimicrobial therapy of unexplained fever in neutropenic patients–guidelines of the Infectious Diseases Working Party (AGIHO) of the German Society of Hematology and Oncology (DGHO), Study Group Interventional Therapy of Unexplained Fever, Arbeitsgemeinschaft Supportivmassnahmen in der Onkologie (ASO) of the Deutsche Krebsgesellschaft (DKG-German Cancer Society) [42]
2003	Diagnosis and antimicrobial therapy of pulmonary infiltrates in febrile neutropenic patients–guidelines of the Infectious Diseases Working Party (AGIHO) of the German Society of Hematology and Oncology (DGHO) [43]
2003	Diagnosis and treatment of documented infections in neutropenic patients–recommendations of the Infectious Diseases Working Party (AGIHO) of the German Society of Hematology and Oncology (DGHO) [44]
2003	Treatment of fungal infections in hematology and oncology - Guidelines of the Infectious Diseases Working Party (AGIHO) of the German Society of Hematology and Oncology (DGHO) [45]
2003	Diagnosis of invasive fungal infections in hematology and oncology - Guidelines of the Infectious Diseases Working Party (AGIHO) of the German Society of Hematology and Oncology (DGHO) [46]
2003	Central venous catheter (CVC)-related infections in neutropenic patients - Guidelines of the Infectious Diseases Working Party (AGIHO) of the German Society of Hematology and Oncology (DGHO) [47]
2003	Sepsis in neutropenia–guidelines of the Infectious Diseases Working Party (AGIHO) of the German Society of Hematology and Oncology (DGHO) [48]
2003	Antimicrobial therapy of febrile complications after high-dose chemo-/radiotherapy and autologous hematopoietic stem cell transplantation–guidelines of the Infectious Diseases Working Party (AGIHO) of the German Society of Hematology and Oncology (DGHO) [49]
2003	Infectious complications after allogeneic stem cell transplantation: epidemiology and interventional therapy strategies–guidelines of the Infectious Diseases Working Party (AGIHO) of the German Society of Hematology and Oncology (DGHO) [50]
2003	Prophylaxis of invasive fungal infections in patients with hematological malignancies and solid tumors–guidelines of the Infectious Diseases Working Party (AGIHO) of the German Society of Hematology and Oncology (DGHO) [51]
2001	Treatment of fungal infections in hematology and oncology. Guidelines of the Working Party on Infections in Hematology and Oncology (AGIHO) of the German Society for Hematology and Oncology (DGHO) [52]

### Main developments within guidelines over three decades

Febrile neutropenia remains an emergency in the treatment of patients with cancer, requiring prompt and evidence-based clinical and antimicrobial management. The AGIHO guidelines are fit for basing local standard operating procedures and adapting to local epidemiology. Major advances AGIHO helped shape are (a) safety and efficacy of antifungal prophylaxis in patients at high risk [53], (b) safety of empiric antibiotic monotherapy in most patients [11], (c) importance of considering local epidemiology in first-line treatment [11], (d) development of pre-emptive strategies for antifungal therapy [54] and (e) development of risk scores and algorithms for a safe outpatient treatment of patients with standard-risk febrile neutropenia [11]. As antimicrobial resistance is an emerging threat, antimicrobial stewardship programs rationalize empiric treatment by applying PEN-FAST criteria to identify patients eligible for penicillin derivatives [55], despite history of allergy treatment according to local resistance profiles, and early discontinuation of empirical antibiotic therapy after 3–5 days of defervescence and clinical recovery irrespective of the neutrophil count [11, 55, 56]. Close clinical monitoring remains the cornerstone in the care of neutropenic patients and that remained the mandatory paradigm over the last 30 years.

#### Prophylaxis

In the first AGIHO guideline in 2003, the only strong recommendation was for fluconazole prophylaxis in allogeneic bone marrow or stem cell transplant recipients [51]. Only afterwards, posaconazole took this position as preferred agent for prophylaxis, preventing invasive fungal diseases IFD and death due to its broader spectrum covering *Aspergillus* and emerging molds [53]. In the most recent update, this has not changed since no new drugs for prophylaxis have become available in almost two decades [12] – a trend that may be broken by the potential licensing of rezafungin as primary prophylaxis in patients undergoing allogeneic HCT [57]. Other antifungal strategies like diagnostic-driven pre-emptive treatment may become more important in antifungal stewardship facing increasing antifungal resistance, but also better access to imaging, specifically PET-CT, and biomarkers [54, 58, 59].

#### Respiratory viral infections

The COVID-19 pandemic highlighted the specific vulnerability of patients with cancer for community-acquired respiratory viral infections. During the pandemic, AGIHO updated guidance in close succession providing physicians with up-to-date evidence-based recommendations [13, 16, 19, 60]. Besides SARS-CoV-2, other respiratory viruses, primarily influenza or respiratory syncytial virus (RSV) cause significant morbidity and mortality in patients with cancer and similar guiding principles apply to diagnosis and therapy [9]. These comprise a high level of awareness and prompt identification of a pathogen allowing early antiviral therapy to pre-empt progression to lower respiratory tract infection [9]. Preventative infection control measures and vaccination strategies against SARS-CoV-2, influenza and RSV are vitally important to protect our vulnerable patient populations [7].

#### Vaccination

Recent major advances in cancer treatment, for example antibody-drug conjugates, chimeric antigen receptor T cells, bispecific antibodies come with previously unknown infection patterns. In some cases, innovative treatments are even associated with specific infections [61, 62]. One example is proteasome inhibitors in multiple myeloma frequently leading to herpes zoster. Another area is treatment of lymphoma with anti‑CD20 antibodies or Bruton tyrosine kinase inhibitors, and especially after anti‑BCMA bispecific antibodies, where infections represent a major threat. The risk posed by some of these infections can be mitigated by vaccination as the earliest and thus most effective preventive measure [7]. However, vaccine protection must be regularly assessed and, if necessary, renewed, especially after severe immunosuppression [7], but vaccinations are heavily underused in our patients [63, 64]. To date, only a few clinical studies on vaccinations specifically evaluate clinical endpoints such as infection rates, sick days or work absenteeism, morbidity, or mortality in cancer patients. In contrast, numerous studies assess antibody formation or T‑cell-mediated immunity as surrogate markers of immune protection. In 2018, the AGIHO published its first guideline on vaccinations in patients with cancer [23], and another update in 2026 focusses specifically on recipients of new cancer treatments [7].

### Contributions to European and global efforts

Since the early 2010 s AGIHO has been a core part of the multi-national, evidence-based clinical practice guidelines of the ECIL (https://ecil-leukaemia.com), along with the European Organization for Research and Treatment of Cancer (EORTC) and the European Society for Blood and Marrow Transplantation (EBMT). The scope of ECIL guidelines, in line with AGIHO guidelines, include empirical antimicrobial treatment, antibacterial and antifungal prophylaxis, specific guidelines for viral or *Pneumocystis* infections as well as guidelines on infections in HCT recipients [65–71]. AGIHO closely interacts with the ID Working Party of the EBMT, current joint commitments are in cytomegalovirus infection [72], invasive fungal diseases [73], and CNS infection [74]. In addition, the IDWP regularly organizes international training courses, which were hosted and supported repeatedly by AGIHO members. From 2019 the AGIHO principles on how to evaluate strength of recommendations and levels of evidence were employed in the Global Guideline Initiative lead by the European Confederation of Medical Mycology (ECMM), which was joined by the Mycoses Study Group Education and Research Consortium (MSGERC), the International Society for Human and Animal Mycology (ISHAM), and the American Society for Microbiology (ASM). These guidelines focus exclusively on fungal infections and were endorsed by more than 100 national and regional societies worldwide [60, 75–80]. Additional collaborations include European Hematology Association (EHA) initiated guideline development [81] and surveys on infection management in cellular therapies and bispecific antibody treatment, ID training in hematology, and antifungal stewardship among hematologists [82]. Together, these efforts highlight AGIHO’s key role in strengthening pan-European initiatives in infections in hematology.

## Clinical Research

While the focus is on guidelines, AGIHO research addresses areas of uncertainty. The studies range from infection control measures to outbreaks [83–85]. Currently enrolling studies are a real-world registry on central venous catheters (CVC). secrecy was initiated in 2013 and includes > 7,100 CVC while spanning 16 academic and non-academic centers. SECRECY enhances understanding of clinical routines [86], provides epidemiological data on CVC-related bloodstream infections [87, 88]. The data set enables registry-based randomized simulation studies [89], and serves generating hypotheses for interventional studies.

FungiScope is an international registry established in 2003 by AGIHO members to create real-world evidence on rare and emerging IFD [90]. Epidemiology of these pathogens hardly allow randomized controlled trials, and many participants from 93 countries facilitates multiple investigator-driven sub-studies. To date, FungiScope holds > 7,200 individual patient courses of rare IFD, enabling a substantial body of peer-reviewed evidence on uncommon and difficult-to-treat IFD, directly informing international treatment guidelines [77, 79, 80, 91–100]. FungiScope registry data served as external matched control for licensing purposes [101]. The registry supports regulatory decision-making and fast-track approvals by the U.S. Food and Drug Administration (FDA) and European Medicines Agency (EMA), accelerating patient access to innovative antifungal treatments [101].

The EHA Specialized Working Group (SWG) “Infections in Hematology” was established in 2017 under the leadership of Austrian and German AGIHO. The close collaboration between AGIHO and the EHA SWG generates new evidence. During the COVID-19 pandemic, AGIHO members together with SEIFEM (Sorveglianza Epidemiologica Infezioni nelle Emopatie – Infection Study Group within the Italian Society of Hematology) supported EPICOVIDEHA (now EPIRESEHA) [102, 103], the largest registry on respiratory viral infections in patients with hematological diseases [38, 102–126], followed by EPIAMLINF on infections in AML, and “HemaShield” on infections after bispecific antibody treatment [127].

In 2023, AGIHO members initiated a study on next generation sequencing (NGS) examining the added value in CNS infection after HCT (ZNMoDiSZ) [128]. This prospectively controlled study employs NGS on cerebrospinal fluid (CSF) samples from patients with overt meningitis or encephalitis to identify pathogens in addition to conventional methods [129, 130].

## Conclusion and outlook

Over the last 30 years the AGIHO supported hematologists and oncologists through 48 guideline documents, and by educational sessions and courses. The members of the AGIHO are enthusiastically continuing this service. Several guidelines and updates are currently in progress, i.e. IFD, CVC-related infections, prophylaxis of bacterial infections, prophylaxis and treatment of infections in cellular therapy. The next generation is integrated through talented young AGIHO clinicians and scientists.

## Data Availability

No datasets were generated or analysed during the current study.
